# DeepWalk-Based Graph Embeddings for miRNA–Disease Association Prediction Using Deep Neural Network

**DOI:** 10.3390/biomedicines13030536

**Published:** 2025-02-20

**Authors:** Jihwan Ha

**Affiliations:** Major of Big Data Convergence, Division of Data Information Science, Pukyong National University, Busan 48513, Republic of Korea; jhha@pknu.ac.kr; Tel.: +82-51-629-4614

**Keywords:** machine learning, DeepWalk, deep neural network, miRNA, disease, miRNA–disease association

## Abstract

**Background:** In recent years, micro ribonucleic acids (miRNAs) have been recognized as key regulators in numerous biological processes, particularly in the development and progression of diseases. As a result, extensive research has focused on uncovering the critical involvement of miRNAs in disease mechanisms to better comprehend the underlying causes of human diseases. Despite these efforts, relying solely on biological experiments to identify miRNA-disease associations is both time-consuming and costly, making it an impractical approach for large-scale studies. **Methods:** In this paper, we propose a novel DeepWalk-based graph embedding method for predicting miRNA–disease association (DWMDA). Using DeepWalk, we extracted meaningful low-dimensional vectors from the miRNA and disease networks. Then, we applied a deep neural network to identify miRNA–disease associations using the low-dimensional vectors of miRNAs and diseases extracted via DeepWalk. **Results:** An ablation study was conducted to assess the proposed graph embedding modules. Furthermore, DWMDA demonstrates exceptional performance in two major cancer case studies (breast and lung), with results based on statistically robust measures, further emphasizing its reliability as a method for identifying associations between miRNAs and diseases. **Conclusions:** We expect that our model will not only facilitate the accurate prediction of disease-associated miRNAs but also serve as a generalizable framework for exploring interactions among various biological entities.

## 1. Introduction

MicroRNAs (miRNAs) are short RNA sequences, usually around 22 nucleotides in length, that are not involved in protein synthesis. These molecules regulate the expression of genes by interacting with the 3′ untranslated regions (UTRs) of target messenger RNAs (mRNAs) [1,2,3,4,5]. This interaction occurs through partial base pairing between the miRNA and the mRNA, which results in the repression or degradation of the target mRNA. In other words, miRNAs play a crucial role in post-transcriptional regulation by influencing how much of a particular protein is produced. They act as fine-tuners of gene expression, ensuring that the right proteins are synthesized at the right time and in the right amounts. The binding of miRNAs to the UTRs of mRNAs is a highly specific process, allowing them to target particular genes and regulate their expression effectively [1,2,3,4,5]. miRNAs perform dual functions: they inhibit protein synthesis by disrupting gene expression and can also act as activators [6]. Since the discovery of the first miRNA, *lin-4*, numerous miRNAs have been identified through advanced high-throughput methods [7,8]. Research has highlighted their critical roles in various biological processes, including aging [9], apoptosis [10], development [11], and cell proliferation [12]. Consequently, identifying miRNAs associated with diseases is crucial both for understanding molecular mechanisms and for diagnosing complex human disorders. Given the high cost and time requirements of laboratory experiments, many studies have focused on designing computational models to predict disease-related miRNAs.

### 1.1. Related Works

Extensive research has suggested that microRNAs (miRNAs) sharing similar functional characteristics are frequently associated with diseases presenting analogous phenotypic traits. This foundational concept is widely employed in predicting miRNA–disease associations (MDAs) [13,14]. Ha et al. introduced a probabilistic matrix factorization model, which incorporates miRNA expression data as implicit feedback, to identify potential MDAs [15]. They further enhanced the model by integrating a disease similarity constraint, resulting in a notable improvement in MDA prediction accuracy [16]. Expanding on this, the authors proposed an innovative model that combines neural collaborative filtering with deep learning techniques to infer miRNA–disease relationships more effectively [17]. Jiang et al. developed a novel framework that leverages a hypergeometric distribution, utilizing a variety of heterogeneous networks involving miRNAs and diseases to identify miRNAs related to specific diseases [18]. Shi et al. took a different approach, designing a random walk framework to identify disease-related miRNAs through a bipartite network [19]. Similarly, Mørk et al. introduced an efficient model that exploits protein associations to link miRNAs and diseases, collecting relevant data from protein–miRNA and protein–disease relationships through text mining [20]. Xu et al. proposed a method for ranking disease-associated miRNAs by incorporating disease gene data and miRNA–target interactions [21]. Xuan et al. employed the k-nearest neighbor algorithm within a constructed network to predict miRNA–disease associations for HDMP, assuming that miRNAs within the same cluster are highly associated with a common disease [22]. Chen et al. presented a novel prediction method, RWRMDA, which uses a random walk algorithm to explore a global miRNA functional similarity network to uncover new MDAs [23].

Machine learning has emerged as a crucial methodology across numerous scientific fields, with bioinformatics standing out as a notably dynamic domain of application. Numerous research groups have utilized machine learning models to uncover miRNA–disease associations, leveraging diverse biological data sources for enhanced predictions [24,25,26]. One such approach was introduced by Chen et al., who utilized k-nearest neighbors (KNN) to predict miRNA–disease associations [27]. Their model integrates heterogeneous biological datasets, enabling the identification of potential miRNAs related to specific diseases. A key feature of their model is the ranking of miRNAs based on disease relevance, which is achieved through a support vector machine (SVM) scoring system. This integration of multiple data types and machine learning algorithms has proven effective in uncovering complex biological relationships. Chen et al. explored hierarchical agglomerative clustering to refine their predictions of miRNA–disease associations, factoring in miRNA–disease bias ratings to improve accuracy [28]. This method builds on the foundation of their earlier work, incorporating clustering techniques to better handle the inherent heterogeneity in biological data. The same research group also proposed a novel method called RLSMDA, which applies a semi-supervised classifier to predict miRNAs that have not yet been associated with known diseases [29]. This model is particularly valuable for expanding the scope of miRNA–disease association prediction by focusing on previously uncharted miRNAs. Xiao et al. introduced a prediction method called GRNMF, which applies non-negative matrix factorization (MF) to various types of omics data [30]. GRNMF excels in identifying both miRNAs that lack known disease associations and diseases that have not yet been linked to any miRNA. By leveraging multiple types of omics data, this model offers a more comprehensive approach to miRNA–disease prediction. Li et al. proposed MCMDA, a matrix factorization framework that updates the adjacency matrix of miRNA–disease associations to improve prediction accuracy [31]. MCMDA’s efficient matrix updating process enables it to better capture complex relationships between miRNAs and diseases, enhancing its predictive power. Chen et al. introduced HGIMDA, a method that integrates multiple similarity measures to construct a comprehensive network for predicting potential miRNA–disease associations [32]. By considering various similarity values, HGIMDA improves the robustness of miRNA–disease predictions, making it a promising tool for further advancing this area of research. Ha et al. introduced a prediction model called PMAMCA, which utilizes matrix factorization (MF) to identify miRNAs associated with diseases [33]. MF, a machine learning technique commonly used in recommendation systems, was applied to capture disease-related miRNAs by incorporating miRNA expression data into the model. This approach allows for the efficient identification of relevant miRNAs, highlighting the versatility of MF in bioinformatics applications. In a similar vein, Chen et al. proposed a method that integrates various similarity measures—miRNA functional similarity, disease semantic similarity, and Gaussian interaction profile kernel similarity—to predict disease-related miRNAs [34]. This model enhances prediction accuracy by combining these diverse sources of information into a unified framework, making it a more comprehensive tool for identifying miRNA–disease associations. Building on this, the same group developed MDHGI, a method that integrates multiple similarity values for improved miRNA–disease association prediction [35]. The inclusion of various similarity metrics strengthens the robustness of the predictions, offering a more reliable way to uncover disease-related miRNAs. Further expanding on this approach, Chen et al. introduced NCMCMDA, a model designed to identify novel disease-related miRNAs. NCMCMDA combines matrix completion algorithms with comprehensive similarity measures, reflecting similarity-based neighborhood constraints to enhance prediction accuracy [36]. By using matrix completion techniques, this model is able to better capture hidden relationships between miRNAs and diseases, making it an effective tool for discovering new associations. Ha et al. employed matrix factorization (MF), a machine learning technique widely used in recommendation systems, to efficiently predict miRNAs linked to diseases. By incorporating similarity information between diseases and miRNAs, their approach significantly enhanced the identification of relevant miRNAs associated with specific diseases [37]. This model showcases how matrix factorization, originally designed for recommendation tasks, can be effectively adapted for complex biological prediction problems. In addition to this, the authors proposed another machine learning model that utilizes metric learning to further refine the process of identifying disease-related miRNAs. Metric learning, which focuses on learning the similarity between data points, allows for more precise predictions by distinguishing between relevant and non-relevant miRNAs based on their similarity to disease profiles [38]. Yu et el.’s proposed method utilizes attributed multi-layer heterogeneous network embedding to learn latent representations of miRNAs and diseases for each association type and predicts the existence of association types for all miRNA–disease pairs by leveraging non-linear characteristics in the network [39]. Ha et al. also presented a machine-learning model (GCNCF) designed to predict disease-related miRNAs by combining graph convolutional neural networks for capturing network structures and feature vectors with neural collaborative filtering to identify miRNA–disease associations [40]. Ning et al. introduced a novel method, AMHMDA, which combines attention-aware multi-view similarity networks with hypergraph learning to predict miRNA–disease associations [41]. The attention-aware mechanism enables the model to focus on the most informative features, while multi-view similarity networks help capture the complex relationships across different data sources. Hypergraph learning, which is capable of handling higher-order relationships between miRNAs and diseases, further improves the model’s predictive power. This innovative approach highlights the increasing sophistication of machine learning methods, integrating multiple data sources and learning strategies to achieve more accurate predictions. Similarly, Jin et al. developed MAMFGAT, a cutting-edge model that integrates adaptive modality fusion with graph attention networks to predict miRNA–disease associations. By combining various similarity networks and association data, MAMFGAT refines the prediction process, offering a more holistic understanding of the interactions between miRNAs and diseases [42]. The use of graph attention networks enables the model to assign different importance to various features, further enhancing its ability to identify significant miRNA–disease relationships. Peng et al. proposed MHCLMDA, an advanced method that utilizes hypergraph contrastive learning alongside variational autoencoders to predict miRNA–disease associations. This model integrates consistent feature representations from multiple views, which helps improve the robustness of the predictions by ensuring that the model captures essential patterns across diverse datasets [43]. By combining hypergraph learning with contrastive learning and autoencoders, MHCLMDA offers a powerful approach to predict miRNA–disease associations, showcasing the potential of combining multiple advanced machine learning techniques to tackle complex biological prediction tasks. Zhao et al. proposed MotifMDA, a novel motif-aware MDA prediction model that leverages high- and low-order structural information from MDA networks [44]. Toprak et al. proposed a computational method utilizing matrix decomposition to predict miRNA–disease associations. They employed nuclear norm minimization to enhance low-rank approximation and extracted key miRNAs associated with breast cancer [45]. Ouyang et al. proposed the HGCLAMIR model, which integrates a hypergraph convolutional network (HGCN) with contrastive learning to enhance high-order relation learning and representation capability. Additionally, they introduced a view-aware attention mechanism and integrated multi-view representation learning to adaptively weight and combine embeddings, ultimately improving miRNA–disease association prediction [46].

The model uses designed motifs to capture network structural patterns and a two-layer hierarchical attention mechanism to learn motif preferences and final embeddings of miRNAs and diseases. These advancements highlight the growing influence of machine learning techniques in bioinformatics, particularly in predicting miRNA–disease associations. By integrating diverse biological data with sophisticated algorithms, researchers have made significant strides in uncovering the complex relationships between miRNAs and diseases. Furthermore, the combination of various algorithms and learning strategies—such as matrix factorization, attention mechanisms, hypergraph learning, and autoencoders—has proven to greatly enhance the accuracy and reliability of miRNA–disease association predictions. This progress is crucial for advancing our understanding of diseases and developing effective therapeutic strategies.

### 1.2. Proposed Study

In this study, we aim to tackle the previously mentioned challenges by developing a neural architecture known as the DeepWalk-based deep neural network method (DWMDA) for predicting miRNA–disease associations. Given that neural networks have demonstrated remarkable proficiency in approximating continuous functions, and deep neural networks (DNNs) have excelled in diverse fields such as speech recognition, computer vision, and various biological applications; it is essential to incorporate DNNs for the task of identifying disease-associated miRNAs. DWMDA outperforms existing methods, achieving impressive areas under the receiver operating characteristic (ROC) curve values (AUCs) of 0.9260 and 0.9101 in global and local leave-one-out cross-validation (LOOCV) frameworks, respectively. In addition, through a comprehensive evaluation using multiple statistical metrics such as AUC, AUPRC, ACC, and MCC, we were able to further substantiate the superiority of our model. These metrics collectively highlight the model’s robust performance, demonstrating its effectiveness across different aspects of classification and generalization. Moreover, we performed a series of comprehensive experiments to qualitatively evaluate the advantages of incorporating low-dimensional miRNA and disease vectors, which were generated using DeepWalk. These experiments provide further evidence of the enhanced predictive power and robustness of the proposed method when leveraging the refined representations of miRNAs and diseases.

The key determinants of accurate miRNA association prediction can be summarized as follows: (1) the robust representation of miRNA–disease associations through the utilization of a Gaussian interaction profile kernel, which improves the fidelity of network modeling; (2) the implementation of a DeepWalk-based approach to derive informative, low-dimensional embeddings from miRNA and disease networks, effectively capturing their latent relationships; and (3) the incorporation of a deep neural network to classify disease-related miRNAs with high precision, leveraging the learned embeddings to enhance predictive reliability.

Our paper is organized as follows: In the Introduction, we provide an overview of the research field, discuss related works, and briefly introduce the methodology proposed in this paper. In Section 2: Materials and Methods, we present a detailed description of the datasets used and the methodologies employed in our approach. Section 3: Results demonstrates the effectiveness of our proposed model through a series of experiments. In Section 4: Discussion, we analyze the results in depth, highlighting the implications and limitations of our work. Finally, in Section 5: Conclusion, we summarize the key findings and suggest directions for future research.

## 2. Materials and Methods

### 2.1. Human miRNA–Disease Association Data

To construct a comprehensive dataset of miRNA–disease associations (MDAs), we integrated information from multiple publicly available databases to ensure data completeness and reliability. The Human MicroRNA Disease Database (HMDD) v3.2 served as the primary source, providing 8968 experimentally validated associations involving 788 unique miRNAs and 374 distinct diseases [47]. In addition, we incorporated data from miR2Disease, a curated repository that systematically compiles 3273 miRNA–disease associations covering 349 miRNAs and 136 diseases [48]. Furthermore, we utilized dbDEMC v2.0, a specialized database focusing on cancer-related miRNAs, which offers detailed annotations for 2224 miRNAs across 36 cancer types [49]. By integrating these datasets, we aimed to construct a robust and diverse MDA dataset that captures a broad spectrum of miRNA–disease interactions. To ensure data consistency and usability, duplicate entries across the datasets were systematically removed. Disease terminology was standardized using the Medical Subject Headings (MeSH) vocabulary to harmonize nomenclature and facilitate downstream analyses. This step was crucial for developing a reliable gold-standard dataset for benchmarking and validation [50]. From the curated and standardized dataset, a binary matrix ($A\in R^{n_{m}\times n_{d}}$) was constructed to capture the relationships between miRNAs and diseases. Each element in the matrix indicates the presence (1) or absence (0) of a validated association. This matrix forms the cornerstone for subsequent computational modeling and machine learning analyses, as detailed in Equation (1).

(1) $$A\left(m_{i},d_{j}\right)=\left\{\begin{array}{c} 1\left(m_{i},d_{j}\right)\in gold-standarddataset\\ 0\left(m_{i},d_{j}\right)\notin gold-standarddataset \end{array}\right.$$

### 2.2. MiRNA Functional Similarity

In a network, edges represent the degree of similarity between pairs of nodes. To model functional relationships among miRNAs, we utilized functional similarity data derived from MISIM, which was incorporated as edge attributes in the miRNA functional similarity network (*FS*) [51]. MISIM calculates pairwise similarity scores that quantify the functional connections between miRNAs based on shared biological pathways, co-regulated target genes, and other functional characteristics. These functional similarity scores, denoted as *FS*(*m*(*i*) and *m*(*j*)), were used to establish the miRNA similarity network. In this network, higher scores signify stronger functional associations between miRNAs.

### 2.3. Disease Semantic Similarity

To estimate similarity values among diseases, we utilized a directed acyclic graph (DAG), which is a type of directed graph where edges have a specific direction and no cycles exist. This structure is well-suited for representing hierarchical relationships, such as those found in disease ontologies. In our approach, the disease DAG for a given node *P* is defined as (*P*, A(*P*), EG(*P*)), where A(*P*) represents the set of ancestor nodes of *P*, capturing its hierarchical context. EG(*P*), on the other hand, denotes the edges connecting parent nodes to their child nodes. These edges are calculated using Equations (2) and (3), which quantify the structural relationships within the DAG and enable similarity assessments between diseases.

(2) $$DV(P)=\sum_{c\in A(P)}P_{P}(c)$$

(3) $$\left\{\begin{array}{ll} P_{P}(c)=1\; & if\;c=P\\ P_{P}(c)=\max\{\Delta_{*}P_{P}(c^{\prime })|c^{\prime }\in children\;of\;c\}\; & if\;c\neq P \end{array}\right.$$

In the equations outlined, ∆ denotes the semantic contribution factor, which measures the increase in semantic association between two diseases as their distance within the semantic structure decreases. This factor is grounded in the assumption that diseases positioned closer together in a directed acyclic graph (DAG) are more likely to share common characteristics. The proposed scoring model is based on this premise, suggesting that a greater overlap of shared elements within the DAG corresponds to a higher degree of similarity between diseases. We introduce SS as the disease semantic similarity matrix, a tool for quantitatively assessing the degree of similarity between pairs of diseases. The semantic similarity between diseases *i* and *j* is explicitly expressed in Equation (4). This methodology offers a systematic and scalable framework for evaluating disease relationships based on their semantic context.

(4) $$SS(d(i),d(j))=\frac{\sum_{t\in A(i)\cap A(j)}\left(P_{i}(t)+P_{j}(t)\right)}{DV(i)+DV(j)}$$

The Gaussian interaction profile (GIP) kernel has been widely utilized in diverse research areas to effectively capture interaction patterns, such as relationships between genes, diseases, and even users in social networks [52,53]. Given its robustness, we adopted the GIP kernel to measure similarity scores between miRNAs and diseases based on available data regarding known miRNA–disease associations. In this context, IP(*m*(*i*)) serves as a profile vector that specifies whether a particular miRNA is linked to a specific disease *d*(*i*). This profile information provides a structured representation of miRNA–disease interactions, enabling the calculation of GIP similarity between any two miRNAs, *m*(*i*) and *m*(*j*), through the following mathematical expression:

(5) $$GS(m(i),\;m(j))=\exp(-r_{l}∥IP(m(i))-IP(m(j)){∥}^{2})$$

In this formulation, GS denotes the Gaussian interaction profile (GIP) kernel similarity, while $r_{m}$ represents a hyperparameter that controls the bandwidth of the kernel (Equation (6)). Following insights drawn from prior research, we fixed $r_{m}^{\prime }$ to 1 as a default setting. Through this approach, we derived similarity measures between diseases based on the GIP framework.

(6) $$r_{l}=\frac{r_{d}^{\prime }}{\frac{1}{n_{l}}\sum_{i=1}^{n_{d}}||IP(m(i)|{|}^{2}}$$

### 2.4. Unified Similarity Framework for miRNAs and Diseases

To construct a comprehensive and robust network that leverages multiple sources of similarity data, we integrated the functional similarity (FS) of miRNAs with their Gaussian interaction profile (GIP) kernel similarity (GS). This combined approach enhances the ability to capture diverse relationships between miRNAs, drawing on both their biological functions and interaction profiles. The unified similarity score (US), which serves as the edge weight in the miRNA similarity network, is computed by merging these two similarity measures [37,40]. This unified score effectively reflects the overall similarity between miRNAs, considering both functional and interaction-based features. The unified similarity score (US), which determines the edge weight in the miRNA similarity network, is calculated as follows:

(7) $${US}_{m}\left(m\left(i\right),m\left(j\right)\right)=\left\{\begin{array}{c} FS\left(m\left(i\right),m\left(j\right)\right),\;\;\;\;\;\;\;\;\;\;\;\;\;\;\;\;\;\;\;\;if\;m\left(i\right)and\;m\left(j\right)\;has\;FS\\ {US}_{m}\left(m\left(i\right),m\left(j\right)\right)\;\;\;\;\;\;\;\;\;\;\;\;\;\;\;\;\;\;\;\;\;\;\;\;\;\;\;\;\;\;\;\;\;\;\;\;\;\;\;\;\;\;\;\;\;otherwise \end{array}\right.$$

Likewise, the overall similarity between diseases can be evaluated as follows:

(8) $${US}_{d}\left(d\left(i\right),d\left(j\right)\right)=\left\{\begin{array}{c} SS\left(d\left(i\right),d\left(j\right)\right),\;\;\;\;\;\;\;\;\;\;\;\;\;\;\;\;\;\;\;\;\;\;\;\;\;\;\;if\;d\left(i\right)and\;d\left(j\right)\;has\;SS\\ {GS}_{d}\left(d\left(i\right),d\left(j\right)\right)\;\;\;\;\;\;\;\;\;\;\;\;\;\;\;\;\;\;\;\;\;\;\;\;\;\;\;\;\;\;\;\;\;\;\;\;\;\;\;\;\;\;\;\;\;\;\;\;\;otherwise \end{array}\right.$$

### 2.5. DeepWalk: A Framework for Node Representation

Graphs and networks are fundamental tools for modeling complex systems, capturing relationships and interactions across various domains such as biology, social sciences, and computer networks. Their structural properties, including centrality, clustering, and connectivity, provide critical insights into the dynamics of interconnected systems. In bioinformatics, graph-based approaches are particularly valuable for representing molecular interactions, uncovering disease pathways, and predicting biomolecular associations. The versatility of graphs in modeling both local and global interactions make them an essential framework for analyzing large-scale, high-dimensional data. As real-world systems become increasingly complex, network science continues to play a pivotal role in understanding the patterns and processes that govern these interconnected phenomena.

**Figure 1:**
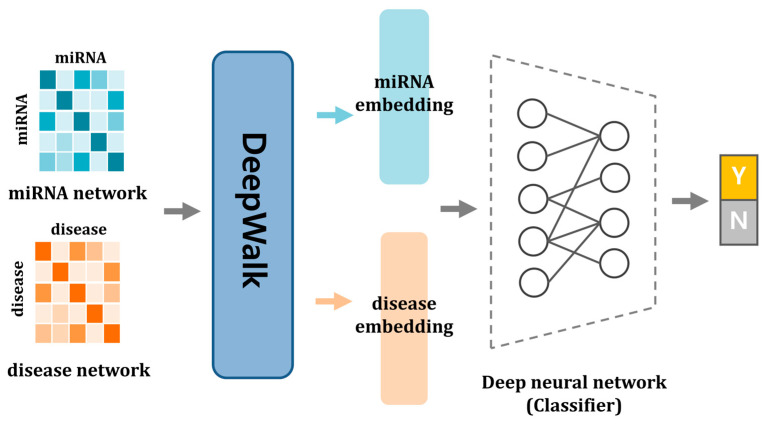
The flowchart of DWMDA. First, miRNA and disease similarity networks are established based on diverse similarity computation techniques. Next, DeepWalk is applied to derive low-dimensional feature embeddings for miRNAs and diseases, effectively capturing the topological and functional characteristics of the networks. Finally, a deep neural network (DNN) model is implemented to identify potential miRNA–disease associations.

The relationships between nodes in a network can be effectively captured using network embedding algorithms. In this study, we utilized DeepWalk to extract behavioral patterns from the miRNA and disease network. DeepWalk is particularly suitable for handling large-scale graphs due to its scalability. Furthermore, it excels in processing sparse datasets, making it a preferred choice for ensuring better generalization in statistical learning tasks. These attributes highlight why DeepWalk was chosen as the embedding algorithm for our approach. DeepWalk transforms node-to-node relationships into dense vector representations. This transformation enables a more structured understanding of the graph latent features. Unlike deep learning-based approaches, DeepWalk is a graph-based embedding method that applies random walks to generate node sequences. These sequences are then processed using the skip-gram model, a shallow neural network commonly used in natural language processing, to learn node embeddings. While skip-gram utilizes a simple neural network, DeepWalk itself is not classified as a deep learning algorithm. The core mechanism of DeepWalk involves treating the graph as input and generating a latent representation as output. This representation can be used to develop models that address specific tasks, such as natural language processing or network analysis [54]. By leveraging local structural information, DeepWalk constructs latent “phrases” for nodes in the network. This is achieved by employing truncated random walks, analogous to generating sentences in natural language from a sequence of words. This integration of graph theory and language modeling offers an efficient and scalable solution for uncovering meaningful patterns in large and sparse networks. By combining the power of random walks and the skip-gram model, DeepWalk effectively captures the topological structure of the graph, enabling robust and interpretable embeddings. Ultimately, this method combines the power of random walks and language modeling to provide a robust framework for understanding complex networks. Algorithm 1 outlines the step-by-step implementation of the DeepWalk algorithm, detailing its process from input graph construction to vector generation and learning. Additionally, the overall workflow of DWMDA is presented in Figure 1.
**Algorithm 1**: Pseudocode of DeepWalk**Input:** G = (V, E): Graph with vertices V and edges E d: Dimension of embedding γ: Number of random walks per node t: Length of each random walk w: Window size for skip-gram model **Output:** Φ: Embedding matrix of size |V| × d Initialize an empty set of sequences, W ← ∅ **for** each node u ∈ V:  **for** I = 1 to γ:   Initialize a random walk sequence R ← u.    **for** j = 1 to t-1:    Let v be a randomly chosen neighbor of the last node in R, with a bias towards   more global exploration (e.g., based on node degree or other graph properties)     Append v to R.    **end for**
   Add sequence R to W. **end for**
**end for**

DeepWalk takes as input a graph G = (V,E), where V represents the set of nodes and E represents the set of edges. Additional hyperparameters include, d (the dimensionality of the embeddings), γ (the number of random walks per node), t (the length of each random walk), and w (the window size for the skip-gram model). The output is Φ, an embedding matrix of size ∣V∣ × d, where each row corresponds to a d-dimensional embedding vector for a node. Random walks are generated to capture the local structure of the graph. For each node u, γ random walks of length t are performed. Each walk is initialized at u and proceeds by selecting a random neighbor at each step. The set of all random walks, denoted as W, serves as input to the skip-gram model. The skip-gram model is trained to optimize the embeddings Φ such that the conditional probability P(v∣u;Φ) of observing a context node v given a target node u is maximized. This probability is modeled using a softmax function, and the overall objective is to minimize the negative log-likelihood across all nodes and their contexts. The learned embeddings Φ capture both the local and global structure of the graph. After training, the embedding matrix Φ is returned, providing a compact representation of the graph nodes in a d-dimensional space. In this study, the dimensionality parameter, denoted as d, is set to 128.

### 2.6. miRNA–Disease Association Prediction by DNN

**Figure 2:**
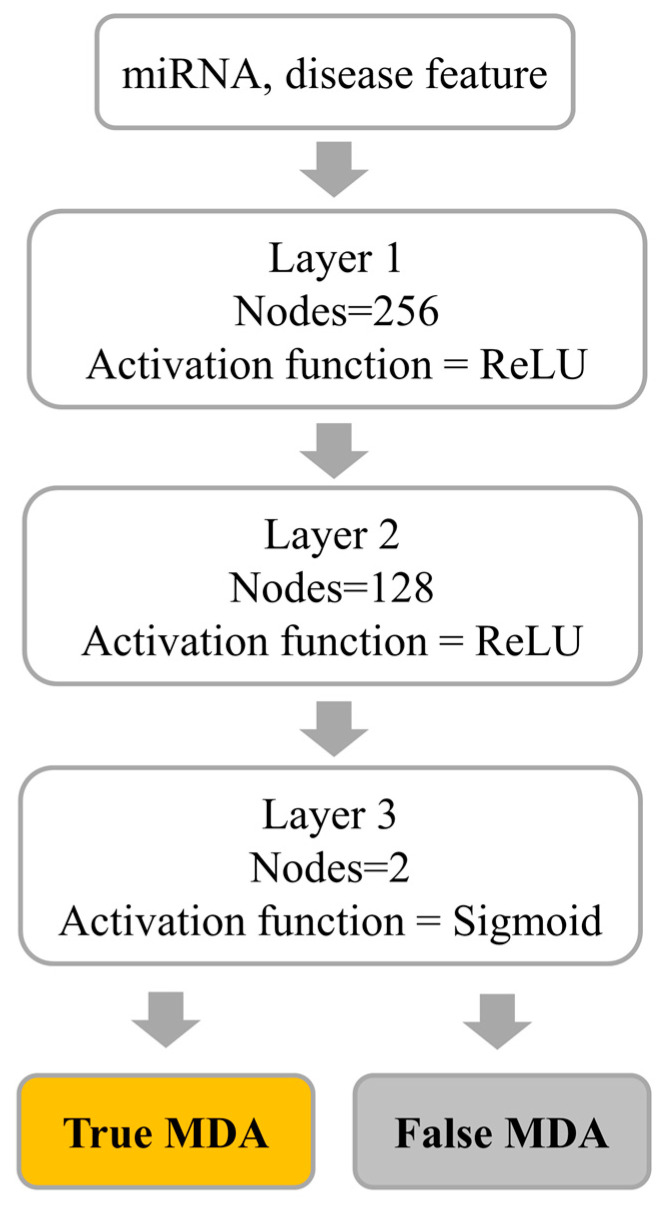
Pipeline for supervised classification of miRNA–disease associations using DeepWalk features.

Using the features extracted from the miRNA and disease network via DeepWalk, we leveraged a deep neural network (DNN) model as a supervised classification method to validate miRNA–disease associations. As illustrated in Figure 2, the DNN architecture consists of three layers, each detailed with the number of neurons, activation functions, and dropout rates. The input to the model comprises miRNA and disease feature vectors derived from DeepWalk, each represented as a 128-dimensional vector. These vectors are concatenated to form a 256-dimensional input vector. The first layer of the DNN contains 256 neurons, activated by the ReLU function, which was chosen for its computational efficiency, sparsity-inducing properties, and ability to address the vanishing gradient problem. Since the task is a binary classification problem, the output layer employs a sigmoid activation function. The binary cross-entropy loss function is used, as it effectively penalizes discrepancies between predicted and actual probabilities, assigning a loss value of 0 for correct predictions and positive values for incorrect ones, with larger errors incurring higher losses. The model optimization is performed using the RMSProp algorithm, which was selected for its effectiveness in handling non-stationary objectives and adaptive learning rate adjustment.

## 3. Results

### 3.1. Performance Measure

To evaluate the performance of DWMDA, we utilized several evaluation metrics, including leave-one-out cross-validation (LOOCV), which is a widely used technique for performance estimation. LOOCV is a special case of n-fold cross-validation, where each data point is used as the test set exactly once. This method is particularly effective for small datasets or cases that require robust validation. LOOCV can be classified into two types: global LOOCV and local LOOCV. In global LOOCV, all diseases are considered simultaneously, whereas local LOOCV focuses on one specific disease at a time. This distinction allows for an evaluation of both broad model performance across multiple diseases and more focused performance on individual diseases. To visually assess the performance of the model under both global and local LOOCV, we plotted Receiver Operating Characteristic (ROC) curves. In the ROC curve, the x-axis represents the false positive rate (FPR), and the y-axis represents the true positive rate (TPR), also known as sensitivity. Sensitivity and specificity were computed using Equations (9) and (10). Sensitivity (TPR) measures the proportion of true positives that were correctly identified, while specificity indicates the proportion of true negatives that were correctly identified.

(9) $$FPR=\frac{FP}{FP+TN}$$

(10) $$TPR=\frac{TP}{TP+FN}$$

Here, TP and FP represent accurately identified and inaccurately identified positive instances, respectively, while TN and FN denote correctly classified and incorrectly predicted negative instances, respectively. Based on the ROC curve, we computed the Area Under the Curve (AUC) score, which serves as a precise measure of the model performance.

(11) $$precision=\frac{TP}{TP+FP}$$

(12) $$recall=\frac{TP}{TP+FN}$$

(13) $$ACC=\frac{TP+TN}{TP+TN+FP+FN}$$

(14) $$MCC=\frac{TP\times TN-FP\times FN}{\sqrt{\left(TP+FP)(TP+FN)(TN+FP)(TN+FN\right)}}$$

Additionally, the Area Under the Precision-Recall Curve (AUPRC) was calculated based on precision and recall (Equations (11) and (12)). The PR curve is especially useful for imbalanced datasets, as it focuses on the performance of the minority class. Unlike the ROC curve, it provides a clearer view of the model’s ability to identify positive instances. Moreover, we also measured performance using accuracy (ACC) and the Matthews correlation coefficient (MCC) (Equations (13) and (14)), which offer a more comprehensive evaluation. While accuracy reflects overall correctness, the MCC provides a balanced assessment, particularly in imbalanced classification tasks.

### 3.2. Comparative Performance Evaluation with Previous Methods

**Figure 3:**
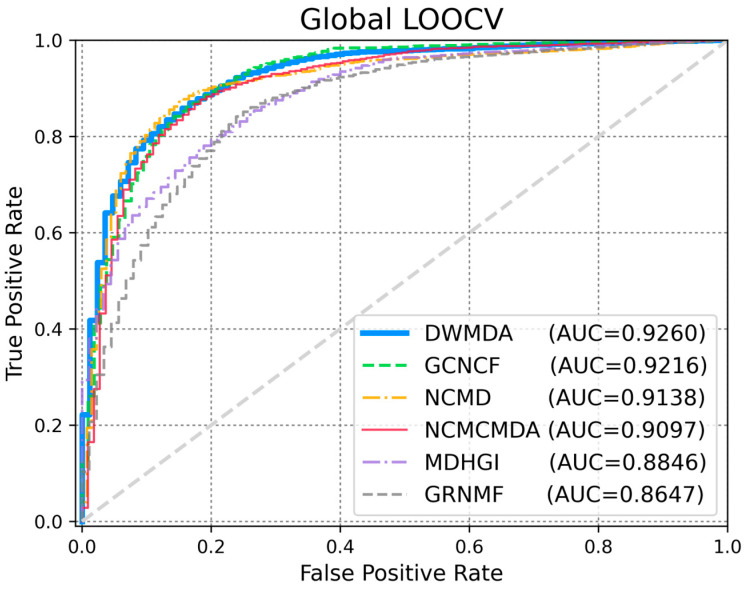
Comparative performance analysis with five established methods using global LOOCV. Among these, DWMDA demonstrated superior effectiveness, achieving the highest AUC score.

**Figure 4:**
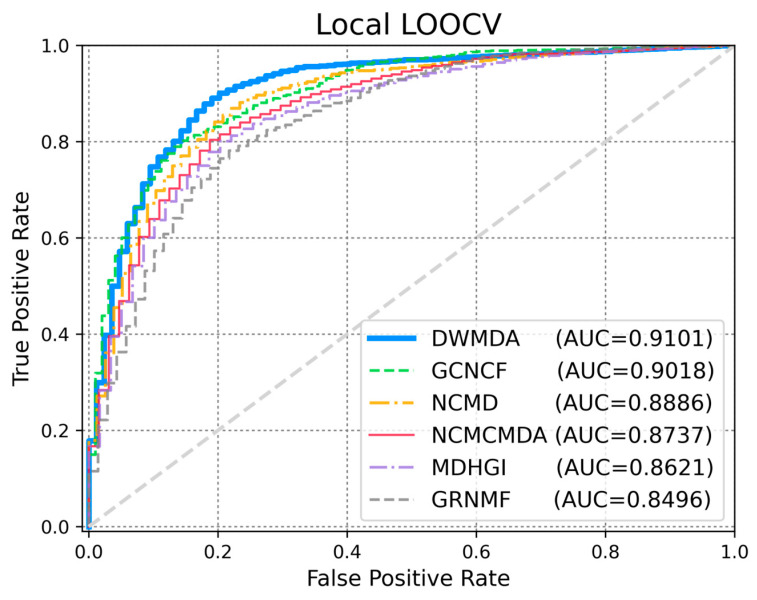
A comparative performance analysis was conducted using local LOOCV with five established methods. Among them, DWMDA exhibited the best performance, attaining the highest AUC score.

To evaluate the performance of DWMDA, we conducted a comparative experiment with several state-of-the-art models, including NCMD [17], GCNCF [40], NCMCMDA [36], MDHGI [35], and GRNMF [30]. These models were selected as benchmark methods because they represent significant advancements in the field and are widely recognized for their effectiveness in predictive tasks. By comparing DWMDA with these models, we aimed to assess its relative performance while ensuring that the comparison was based on similar techniques and approaches, thus providing a robust evaluation of DWMDA’s effectiveness in disease prediction tasks. Based on the global LOOCV (Figure 3), DWMDA achieved a meaningful AUC score of 0.9260, which was superior to those of GCNCF (0.9216), NCMD (0.9138), NCMCMDA (0.9097), MDHGI (0.8846), and GRNMF (0.8647), demonstrating its strong generalization ability across various diseases. We used local LOOCV to demonstrate the performance of DWMDA in identifying novel disease-related miRNAs, which is essential for advancing disease-specific biomarker discovery. As shown in Figure 4, DWMDA obtained an AUC score of 0.9101, indicating that it performed better than GCNCF (0.9018), NCMD (0.8886), NCMCMDA (0.8737), MDHGI (0.8621), and GRNMF (0.8496). This result reinforces the model potential to enhance the understanding of disease mechanisms at the molecular level. Moreover, the model performance was evaluated quantitatively using AUPRC, accuracy (ACC), and the Matthews correlation coefficient (MCC), which offer a more holistic view of its performance. As summarized in Table 1 and Table 2, we demonstrated that DWMDA consistently outperformed the other methods, providing compelling evidence of its superiority based on a wide range of evaluation metrics [40].

### 3.3. Ablation Analysis

**Figure 5:**
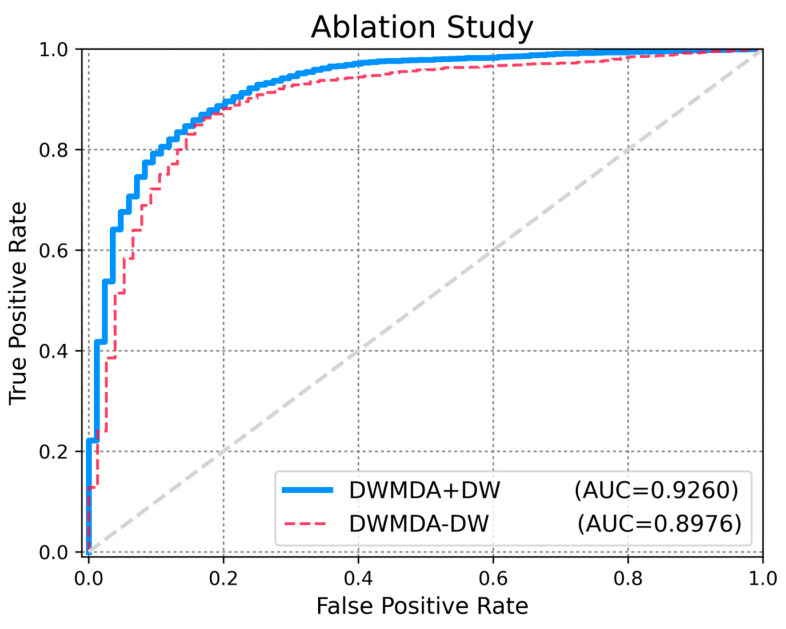
Performance Comparison with and without DeepWalk in DWMDA (AUC).

**Figure 6:**
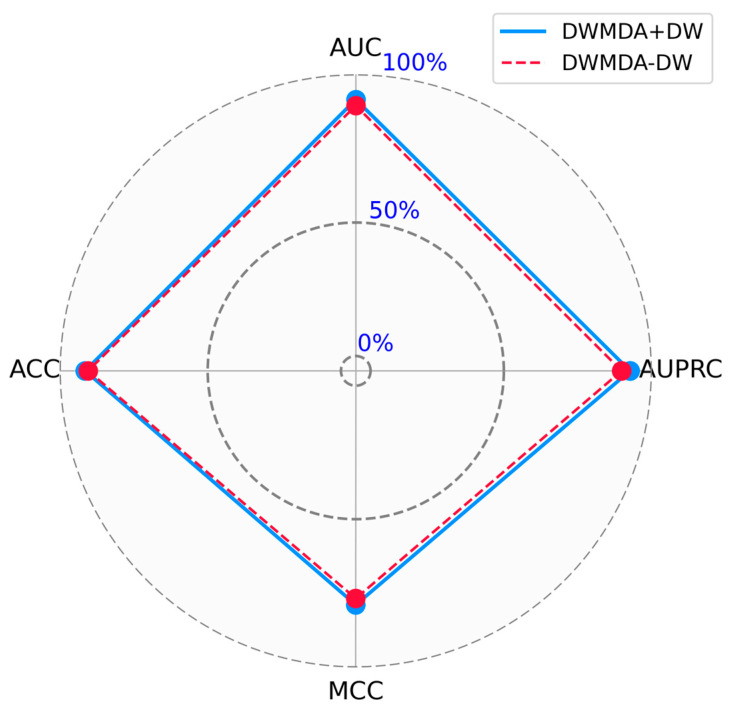
Ablation study for performance evaluation using comprehensive statistical metrics (ACC, MCC, AUPRC, AUC).

Ablation studies are an essential method for evaluating the role of individual components in a machine learning model. By selectively removing or altering specific parts of the model and measuring the resulting performance changes, researchers can determine the relative importance and functionality of each component. This systematic approach not only enhances our understanding of the model’s internal mechanisms but also helps identify critical elements that contribute to its success. Performing ablation studies is a valuable step in optimizing model design, improving efficiency, and ensuring robust interpretability. A key component of our proposed method is the use of DeepWalk to extract low-dimensional representations of diseases and miRNAs from the graph structure. To evaluate the significance of this component, we conducted a comparative analysis of our model with (DWMDA + DW) and without (DWMDA − DW) the integration of the DeepWalk module. The results demonstrate the critical role of DeepWalk in enhancing model performance, underscoring its contribution to capturing meaningful relationships within the graph. As a result, we confirmed that the use of DeepWalk led to improved performance in terms of various statistical metrics, highlighting its effectiveness in enhancing the predictive capability of our model (Figure 5 and Figure 6). This ablation study validates the effectiveness of incorporating DeepWalk in our approach and highlights its importance in achieving accurate predictions (Table 3).

### 3.4. Case Studies

To further validate the performance of DWMDA, we conducted analyses on two prevalent human cancers. Among them, breast cancer (BC) is one of the most frequently diagnosed malignancies in women and remains a leading cause of cancer-related mortality worldwide [55]. Extensive research has demonstrated that miRNAs play a pivotal role in the pathogenesis and progression of BC. For instance, miR-202 and miR-718 have been reported to exhibit elevated expression levels in BC patients, highlighting their potential as early diagnostic biomarkers [56]. Accordingly, we performed a case study to evaluate whether the top-ranked candidates identified by DWMDA are associated with BC. As summarized in Table 4, all highly ranked candidates were confirmed to have significant relevance to BC when validated against the gold standard dataset.

To further explore the applicability of DWMDA, we extended our investigation to lung cancer (LC), a leading cause of cancer-related deaths worldwide that is characterized by the formation of malignant tumors in the lungs [57]. Tobacco smoking has been well-established as the predominant risk factor for LC, although other genetic and environmental factors also contribute to its onset. Notably, miRNAs have been extensively studied for their critical roles in LC progression and regulation [58]. Using the DWMDA framework, we prioritized the top 50 candidate miRNAs based on their predicted associations with LC. Validation against the gold-standard datasets revealed that all predicted miRNAs were closely linked to LC, as detailed in Table 5. These findings underscore the robustness of DWMDA in accurately identifying biomarkers associated with complex diseases.

### 3.5. Pathway Analysis

Pathway analysis represents a powerful functional enrichment approach that sheds light on the molecular interactions and biological mechanisms underlying the expression profiles of differentially expressed genes and proteins. Given that our model was designed to predict disease-associated miRNAs and facilitate insights into their biological activities, we conducted a comprehensive pathway analysis focusing on their predicted targets. To achieve this, we employed DIANA-miRPath v3.0, a widely utilized web-based tool that provides detailed information on miRNA-regulated pathways and their functional roles [59]. Specifically, DIANA-miRPath v3.0 was used to explore the biological relevance of miRNA candidates associated with breast cancer (BC). As summarized in Table 6, the majority of the identified functions exhibited strong associations with BC-related pathways, underscoring the predictive accuracy of our model. The Hippo signaling pathway has emerged as a critical regulator of cell proliferation and apoptosis, both of which significantly influence breast cancer (BC) pathogenesis [60]. This pathway, essential for organ development and wound healing, is deregulated in BC, affecting tumorigenesis, prognosis, and treatment. Targeting its key components, such as YAP1/TAZ and TEAD transcription factors, offers promising therapeutic potential. Similarly, TGF-β signaling transitions from a tumor suppressor in early stages of BC to promoting invasion, metastasis, and malignant progression in later stages [61]. It plays a pivotal role in modifying the tumor microenvironment and facilitating bone and lung metastases, positioning it as another valuable target for treatment strategies. Additionally, while p53 mutations are less frequent in BC compared to other tumors, they are associated with aggressive disease and poor survival [62]. Alterations in p53 regulators and downstream targets also impact BC cases with wild-type p53, underscoring the importance of the pathway in diagnosis, prognosis, and treatment. These findings collectively highlight the pivotal roles in breast cancer progression, providing strong evidence for their potential as key therapeutic and diagnostic targets to improve patient outcomes.

## 4. Discussion

As the volume and complexity of heterogeneous networks continue to expand exponentially, deriving meaningful embeddings from such networks using machine learning has become a critical challenge. To address this issue, the present study focused on two key aspects to ensure comprehensive analysis and validation: (1) creating compact, low-dimensional representations for nodes within the network and (2) capturing the intricate semantics behind the relationships between nodes, such as predicting associations between microRNAs (miRNAs) and diseases. This study is inspired by the observation that networks inherently encode both the structural roles of nodes and their attributes. Building on this, we approach the miRNA–disease association prediction problem by proposing a novel framework called DeepWalk-based miRNA–disease Association (DWMDA). This method integrates diverse biological similarities to construct miRNA and disease-specific networks. For miRNAs, functional similarity measures were utilized, while diseases were represented using semantic similarity and Gaussian interaction profile kernels. These well-established similarity measures provide a robust foundation for constructing reliable networks, ensuring consistency with prior biological research. These similarities collectively capture key biological and clinical insights into miRNAs and diseases. Then, the proposed method employs DeepWalk, a widely used network embedding algorithm, to generate low-dimensional vector representations that effectively preserve the topological structure and roles of nodes. Finally, a deep neural network was successfully utilized to identify miRNAs associated with diseases in an efficient manner. Experimental results demonstrated that DWMDA outperformed existing state-of-the-art models across multiple evaluation metrics, showcasing its ability to uncover hidden patterns in biological networks with greater accuracy. These findings validate the effectiveness of our model in handling the complexity of miRNA–disease association prediction.

However, we acknowledge certain limitations and areas for future improvement. While our method focuses on enhancing prediction accuracy, the underlying graph construction relies on widely adopted similarity measures, such as MISIM for miRNA functional similarity and DAG for disease semantic similarity. Although these are well-established and biologically validated approaches, they do not introduce new computational logic or novel biological hypotheses. To address this, future research could explore alternative similarity metrics or novel strategies for network construction, incorporating additional biological knowledge, such as miRNA-target gene interactions, expression profiles, or environmental influences. Despite the promising results, there remains considerable potential for further enhancement. Integrating more comprehensive and diverse biological datasets, such as miRNA-target gene interactions or environmental influences, could provide deeper contextual insights and significantly improve model performance. Additionally, adopting more advanced machine learning techniques, rooted in solid mathematical frameworks, could refine the model’s ability to incorporate supplementary information, including the intrinsic properties of miRNAs and diseases. This would enable more accurate and reliable predictions in the future. Furthermore, exploring the use of multi-omics data integration and temporal aspects of disease progression could further strengthen the model’s predictive power, offering a more holistic understanding of disease mechanisms and miRNA involvement. Ultimately, continued advancements in data quality, model complexity, and computational techniques will be essential for pushing the boundaries of miRNA–disease association studies and improving the accuracy of predictions.

## 5. Conclusions

In this study, we introduced DWMDA, a DeepWalk-based miRNA–disease association prediction model, which integrates biological similarity networks with deep learning techniques. By leveraging network embeddings and deep neural networks, our method effectively captures complex biological interactions, leading to state-of-the-art performance in miRNA–disease association prediction. The results confirm that our model outperforms existing approaches across various evaluation metrics, demonstrating its robustness and effectiveness. While our primary focus was on improving predictive performance, we acknowledge that future research could further enhance graph construction methodologies by developing novel similarity measures and incorporating additional biological data sources. Moving forward, we aim to explore new similarity metrics and multi-omics data integration to refine network construction. Additionally, the adoption of more advanced deep learning architectures will enable further improvements in predictive accuracy. By addressing these directions, we hope to enhance the biological interpretability and practical impact of miRNA–disease association studies, ultimately contributing to advancements in biomedical research.

## Figures and Tables

**Table 1:** Comprehensive performance comparison using global LOOCV based on various metrics.

Method	AUC	AUPRC	ACC	MCC
DWMDA	0.9260	0.9148	0.9136	0.7887
GCNCF	0.9216	0.9124	0.9006	0.7819
NCMD	0.9138	0.8602	0.8734	0.7514
NCMCMDA	0.9097	0.8678	0.8613	0.7492
MDHGI	0.8846	0.8281	0.8265	0.7252
GRNMF	0.8647	0.8365	0.8209	0.7216

**Table 2:** Extensive performance analysis via local LOOCV considering multiple metrics.

Method	AUC	AUPRC	ACC	MCC
DWMDA	0.9101	0.8943	0.8912	0.7642
GCNCF	0.9018	0.8931	0.8862	0.7547
NCMD	0.8886	0.8468	0.8504	0.7269
NCMCMDA	0.8737	0.8562	0.8372	0.7206
MDHGI	0.8621	0.8117	0.8062	0.7186
GRNMF	0.8496	0.8323	0.7876	0.6988

**Table 3:** Ablation study for performance evaluation using comprehensive statistical metrics.

Method	AUC	AUPRC	ACC	MCC
DWMDA + DW	0.9260	0.9148	0.9136	0.7887
DWMDA − DW	0.8976	0.8942	0.9038	0.7673

**Table 4:** Top-50 breast cancer-related miRNAs predicted by DWMDA and their evidences. All 50 miRNAs were proved to be associated with breast cancer.

Rank	Name	Evidence	Rank	Name	Evidence
1	hsa-mir-618	hmdd, dbDEMC	26	hsa-mir-98	hmdd, miR2disease, dbDEMC
2	hsa-mir-217	dbDEMC,	27	hsa-mir-485-5p	dbDEMC
3	hsa-mir-377	hmdd, dbDEMC	28	hsa-mir-383	hmdd, dbDEMC
4	hsa-mir-145-5p	dbDEMC,	29	hsa-mir-485	hmdd
5	hsa-mir-218-2	hmdd	30	hsa-mir-7-2	hmdd
6	hsa-mir-539	dbDEMC,	31	hsa-mir-27b	hmdd, dbDEMC,
7	hsa-mir-23a	hmdd, dbDEMC	32	hsa-mir-124-3	hmdd
8	hsa-mir-199b-5p	dbDEMC	33	hsa-mir-876-5p	dbDEMC,
9	hsa-mir-320c-2	hmdd	34	hsa-mir-150	hmdd, dbDEMC,
10	hsa-mir-148a	hmdd, miR2disease, dbDEMC	35	hsa-mir-410-3p	dbDEMC,
11	hsa-mir-588	dbDEMC,	36	hsa-mir-502	hmdd
12	hsa-mir-92a	hmdd, dbDEMC,	37	hsa-mir-194-2	hmdd
13	hsa-mir-505	hmdd, dbDEMC,	38	hsa-mir-1179	dbDEMC,
14	hsa-mir-542	hmdd	39	hsa-mir-33a	hmdd, dbDEMC,
15	hsa-mir-140-5p	dbDEMC,	40	hsa-mir-584	hmdd, dbDEMC
16	hsa-mir-34	hmdd	41	hsa-mir-16	hmdd, dbDEMC,
17	hsa-mir-492	hmdd, dbDEMC	42	hsa-mir-92a-3p	dbDEMC,
18	hsa-mir-367	hmdd, dbDEMC	43	hsa-mir-320c-1	hmdd
19	hsa-mir-18a	hmdd, miR2disease, dbDEMC,	44	hsa-mir-409-3p	dbDEMC,
20	hsa-mir-15a	hmdd, dbDEMC,	45	hsa-mir-92a-2	hmdd
21	hsa-mir-517a	dbDEMC,	46	hsa-mir-19b-3p	dbDEMC,
22	hsa-mir-26a-1	hmdd	47	hsa-mir-18b	hmdd, dbDEMC,
23	hsa-mir-30c	hmdd, dbDEMC,	48	hsa-let-7b	hmdd, dbDEMC,
24	hsa-mir-140	hmdd,	49	hsa-mir-526a-2	hmdd
25	hsa-mir-4306	hmdd, dbDEMC	50	hsa-mir-499	hmdd

**Table 5:** Top-50 lung cancer-related miRNAs predicted by DWMDA and their evidences. All 50 miRNAs were proved to be associated with lung cancer.

Rank	Name	Evidence	Rank	Name	Evidence
1	hsa-mir-138-1	hmdd	26	hsa-mir-127	hmdd,
2	hsa-mir-1269	dbDEMC	27	hsa-mir-23a	hmdd, dbDEMC
3	hsa-mir-92-1	hmdd	28	hsa-mir-520f	dbDEMC
4	hsa-mir-451a	hmdd, dbDEMC	29	hsa-mir-192	hmdd, miR2disease, dbDEMC
5	hsa-let-7a-2	hmdd, miR2disease	30	hsa-mir-24-2	hmdd, miR2disease
6	hsa-mir-130b	hmdd, dbDEMC	31	hsa-mir-410	hmdd, dbDEMC
7	hsa-mir-19	hmdd	32	hsa-mir-375	hmdd, dbDEMC
8	hsa-mir-495	hmdd, dbDEMC	33	hsa-mir-330-3p	dbDEMC
9	hsa-mir-218	hmdd, miR2disease, dbDEMC	34	hsa-mir-7	hmdd, miR2disease, dbDEMC
10	hsa-mir-548j	hmdd	35	hsa-mir-493	hmdd, dbDEMC
11	hsa-mir-17	hmdd, dbDEMC	36	hsa-mir-215	hmdd, dbDEMC
12	hsa-mir-34	hmdd	37	hsa-mir-30e	hmdd, dbDEMC
13	hsa-mir-30b	hmdd, dbDEMC	38	hsa-mir-301b	hmdd, dbDEMC,
14	hsa-mir-190b	hmdd, dbDEMC	39	hsa-mir-103	hmdd, dbDEMC
15	hsa-mir-185	hmdd, dbDEMC	40	hsa-mir-216a	hmdd, dbDEMC
16	hsa-mir-19b	hmdd, dbDEMC	41	hsa-mir-299	hmdd
17	hsa-mir-372	hmdd, dbDEMC	42	hsa-mir-450	hmdd, dbDEMC
18	hsa-mir-99b	dbDEMC,	43	hsa-mir-124-3	hmdd
19	hsa-mir-29c	hmdd, miR2disease, dbDEMC	44	hsa-mir-135a-2	hmdd
20	hsa-mir-98	hmdd, miR2disease, dbDEMC	45	hsa-mir-136	hmdd, dbDEMC
21	hsa-mir-206	hmdd, dbDEMC	46	hsa-mir-768	hmdd
22	hsa-mir-21	hmdd, miR2disease, dbDEMC	47	hsa-mir-1	hmdd, miR2disease, dbDEMC,
23	hsa-mir-125a-5p	miR2disease, dbDEMC	48	hsa-mir-3666	dbDEMC
24	hsa-mir-34a	hmdd, dbDEMC	49	hsa-mir-346	hmdd, dbDEMC
25	hsa-mir-196a-1	hmdd	50	hsa-mir-1246	dbDEMC

**Table 6:** Enrichment results for breast cancer-related miRNAs.

KEGG Pathway	*p*-Value
Hippo signaling pathway	1.51 × 10^−10^
TGF-beta signaling pathway	9.45272 × 10^−7^
Renal cell carcinoma	2.16504 × 10^−6^
Pathways in cancer	4.67884 × 10^−5^
Cell cycle	0.000194278
p53 signaling pathway	0.001730424
Pancreatic cancer	0.004375636
Glioma	0.007105886
Non-small cell lung cancer	0.013434261
mTOR signaling pathway	0.015484188
Insulin signaling pathway	0.027279607

## Data Availability

The data presented in this study are openly available in [HMDD v3.0] at [doi: 10.1093/nar/gky1010], reference number [47].
